# Mediation effect of pregnancy-induced hypertension on the association between assisted reproductive technology and adverse neonatal outcomes: a population-based study

**DOI:** 10.1186/s12884-023-05694-3

**Published:** 2023-05-25

**Authors:** Luying Wang, Linbo Cheng, Shimao Zhang, Mi Su, Ying Jin, Dan Luo

**Affiliations:** Department of Obstetrics, Chengdu Women’s and Children’s Central Hospital, School of Medicine, University of Electronic Science and Technology of China, No.1617 Riyue Avenue, Qingyang District, Chengdu, 611731 P.R. China

**Keywords:** Assisted reproductive technology, Pregnancy-induced hypertension, Adverse neonatal outcomes, Mediation effect, National Vital Statistics System

## Abstract

**Background:**

Assisted reproductive technology (ART) has been widely used in the treatment of infertility, and is associated with adverse maternal and neonatal outcomes. However, the potential pathways by which ART affects adverse neonatal outcomes are unclear. We aimed to investigate the role of pregnancy-induced hypertension (PIH) in the association between ART and adverse neonatal outcomes.

**Methods:**

Adult women (aged ≥ 18 years) with a singleton pregnancy in the National Vital Statistics System (NVSS) 2020 were enrolled in this retrospective cohort study. Study outcomes were adverse neonatal outcomes, including premature birth, low birth weight, and admission to the neonatal intensive care unit (NICU). Logistic regression models were utilized to investigate the association between ART, PIH, and adverse neonatal outcomes, expressed as odds ratio (OR) and 95% confidence interval (CI). The distribution-of-the-product method was used to explore whether there was a mediating effect of PIH between ART and adverse neonatal outcomes, and the 95% CI of the distribution-of-the-product did not contain 0 indicating a mediating effect.

**Results:**

This study included 2,824,418 women, of whom 35,020 (1.24%) women used ART, 239,588 (8.48%) women had PIH, and 424,741 (15.04%) neonates had any adverse neonatal outcomes. The use of ART was associated with higher odds of PIH (OR = 1.42; 95%CI: 1.37–1.46) and any adverse neonatal outcomes (OR = 1.47; 95%CI: 1.43–1.51). The distribution-of-the-product was 0.31 (95%CI: 0.28–0.34), and 8.51% of the association between ART and adverse neonatal outcomes was mediated through PIH. Among different adverse neonatal outcomes, PIH mediated 29.17% of the association between ART and low birth weight, 9.37% of the association between ART and premature birth, and 12.20% of the association between ART and NICU admission. The mediating effect of PIH was found in women of different ages (< 35 years and ≥ 35 years) and parities (primipara and multipara).

**Conclusion:**

This study supports a mediating role for PIH in the association between ART and adverse neonatal outcomes. Further studies are needed to determine the mechanisms by which AR affects PIH so that interventions to reduce PIH can be developed to reduce adverse neonatal outcomes associated with ART.

**Supplementary Information:**

The online version contains supplementary material available at 10.1186/s12884-023-05694-3.

## Background

Infertility is defined by the World Health Organization as the failure to achieve a pregnancy after 12 months or more of regular unprotected sexual intercourse, this definition is also supported by the American Medical Association, the European Society for Human Reproduction and Embryology, the International Committee for Monitoring Assisted Reproductive Technologies, and the American Society for Reproductive Medicine [1]. Infertility afflicts many couples worldwide, and the prevalence of infertility varies widely from 3 to 30% in different regions [2–4]. Assisted reproductive technology (ART) is a highly effective artificial fertilization treatment that has been widely used in the last decades for couples with infertility or other causes [5]. The use of ART is growing rapidly worldwide due to the increasing prevalence of infertility and the delayed timing of childbirth [6]. Chambers et al. used data from national and regional ART registries (containing 76 countries and 2,746 ART centers) to show that there were more than 439,039 births among 1,929,905 ART patients in 2014 [7]. However, some non-physiological interventions during ART may affect the overall environment of pregnancy, interfere with gametogenesis or embryonic development, and adversely affect maternal and neonatal outcomes [8, 9]. Several studies have reported that ART was related to higher odds of gestational diabetes, pregnancy-induced hypertension (PIH), placental abnormalities, low birth weight, preterm birth, and neonatal mortality [10–13].

When compared with spontaneous conception, patients with single and multiple pregnancies using ART are at higher risk of PIH [10, 14, 15]. PIH is one of the leading causes of maternal and neonatal adverse outcomes during pregnancy, including gestational hypertension and pre-eclampsia/eclampsia [16]. PIH has been shown to be associated with higher odds of preterm birth, low birth weight, and small gestational age [17–19]. PIH may play a potential role in adverse neonatal outcomes caused by ART. The mechanisms underlying the effects of ART on adverse neonatal outcomes remain uncertain. Identifying, quantifying, and understanding the potential pathways for the association between ART and adverse neonatal outcomes could provide more clinical insights into ART. Stern et al. reported that PIH may play a mediating effect in the association between ART and premature birth [20]. However, whether PIH plays a mediating role in other ART-induced adverse neonatal outcomes is unclear.

We hypothesized that PIH plays a mediating role in the associations between ART and different adverse neonatal outcomes. In this study, we aimed to use data from a large population sample to investigate the association between ART, PIH, and different adverse neonatal outcomes (including premature birth, low birth weight, and admission to the neonatal intensive care unit).

## Methods

### Study design and populations

We conducted a retrospective cohort study based on maternal and neonatal data from the National Vital Statistics System (NVSS) 2020 in the United States (U.S.). The NVSS database is a national open-access database for collecting information on vital events including births, deaths, marriages, divorces, and fetal deaths, established by the National Center for Health Statistics (NCHS) of the Centers for Disease Control and Prevention (CDC) in collaboration with all U.S. states. Detailed description and data access to the NVSS database is available on the official website (https://www.cdc.gov/nchs/nvss/about_nvss.htm). For this analysis, we included women (1) aged ≥ 18 years old; (2) had a singleton pregnancy; (3) gestational age was recorded as ≥ 20 and < 45 weeks; (4) did not have pre-pregnancy hypertension; (5) were evaluated for neonatal outcomes; and (6) with information on whether they use ART. Women with multifetal pregnancies or stillbirths and missing other important covariates such as smoking status and pre-pregnancy body mass index (BMI) were excluded. This study was exempt from approval by the Institutional Review Board of Chengdu Women’s and Children’s Central Hospital because the de-identified data for this retrospective study were obtained from the public NVSS database.

### Outcomes and definition

The outcomes of this study were adverse neonatal outcomes in a live birth. The adverse neonatal outcomes involved were premature birth (defined as gestational age < 37 weeks), low birth weight (defined as birth weight < 2,500 g), and admission to the neonatal intensive care unit (NICU). Live birth is defined as every product of conception that gives a sign of life after birth, regardless of the length of the pregnancy. ART is defined as any fertility treatment involving laboratory handling of eggs or embryosfor the purpose of establishing a pregnancy. PIH was defined as gestational hypertension or eclampsia that occurs during pregnancy [16]. In the U.S. during the studied period [16], gestational hypertension is defined as a systolic blood pressure of 140 mm Hg or more or a diastolic blood pressure of 90 mm Hg or more, or both, on two occasions at least 4 h apart after 20 weeks of gestation in a woman with a previously normal blood pressure. Eclampsia is defined as hypertension with proteinuria with generalized seizures or coma, that may include pathologic edema. Information on ART, PIH, and adverse neonatal outcomes was collected directly from the medical record by the facility worksheet.

### Covariates

Demographics, medical history, antenatal characteristics, and previous pregnancy data were collected. The detailed covariates were maternal age at delivery, race of mother (white, black, Asian, others), educational level of mother (less than high school, high school, bachelor or above, unknown), marital status (married, unmarried, unknown), age of father, race of father (white, black, Asian, others, unknown), educational level of father (less than high school, high school, bachelor or above, unknown), smoking before pregnancy (yes, no), smoking during pregnancy (yes, no), total number of prenatal care visits for this pregnancy, pre-pregnancy BMI, pre-pregnancy chronic diabetes (yes, no), gestational diabetes (yes, no), clinical chorioamnionitis or maternal fever during labor (yes, no), previous preterm births (yes, no), previous cesarean section (yes, no), parity (primipara, multipara), gestational weight gain, and gestational age. Smoking before or during pregnancy was collected using the mother’s worksheet. Information on maternal demographic characteristics was directly obtained from the mother’s worksheet. Maternal and neonatal medical and health information was extracted from the facilities worksheet completed by hospital staff.

### Statistical analysis

Descriptive statistics were presented on population characteristics based on maternal use and non-use of ART. Continuous variables were described as mean ± standard deviation (SD) or median and quartile [M (Q1, Q3)], and the comparison between groups was analyzed by t-test or Mann-Whitney U rank-sum test. Categorical variables were expressed as numbers and percentages [n (%)], and the chi-square test (χ^2^) or rank-sum test was used for comparison between groups.

To represent missing values, we added a category “unknown” to the variable marital status, education level of mother, education level of father, and race of father. All other variables were complete. With any adverse neonatal outcomes, premature birth, low birth weight, and admission to NICU as outcome variables, the univariate logistic regression model was used to screen confounders that might affect these outcomes (Supplement Tables 1, 2, 3 and 4). Univariate and multivariate logistic regression models were utilized to investigate the association of ART with PIH and different adverse neonatal outcomes. Odds ratio (OR) and 95% confidence interval (CI) were used to assess the association. The distribution-of-the-product method was used to explore whether there was a mediating effect of PIH between ART and the risk of adverse neonatal outcomes [21, 22]. The 95% CI for the distribution-of-the-product was calculated using the RMediation package of R software. If the 95%CI of the distribution-of-the-product does not contain 0, which indicates a mediating effect. Model 1 was a univariate logistic regression model; model 2 was a multivariate logistic regression model adjusted for all confounders; model 3 was further adjusted for PIH (intermediate variable) based on model 2. The percentages of mediated explained by PIH were calculated using the formula (OR1 - OR2) / (OR1–1) * 100 [23–25], where OR1 of model 2 (represents total effect) and OR2 of model 3 (represents direct effect). Interaction analyses of the effect of ART and PIH on adverse neonatal outcomes were performed to exclude the interaction between ART and PIH. Subgroup analysis was conducted according to maternal age (< 35 years, ≥ 35 years) and parity (primipara, multipara).

**Table 1:** Maternal and neonatal characteristics

Variables	Total (n = 2,824,418)	SC (n = 2,789,398)	ART (n = 35,020)	*P*
Maternal age at delivery, years, Mean ± SD	29.61 ± 5.54	29.53 ± 5.50	35.84 ± 4.87	< 0.001
Age subgroups, n (%)				< 0.001
<35 years	2,263,460 (80.14)	2,249,290 (80.64)	14,170 (40.46)	
≥35years	560,958 (19.86)	540,108 (19.36)	20,850 (59.54)	
Race of mother, n (%)				< 0.001
White	2,156,689 (76.36)	2,129,552 (76.34)	27,137 (77.49)	
Black	357,342 (12.65)	355,554 (12.75)	1,788 (5.11)	
Others	105,102 (3.72)	104,379 (3.74)	723 (2.06)	
Asian	205,285 (7.27)	199,913 (7.17)	5,372 (15.34)	
Educational level of mother, n (%)				< 0.001
Less than high school	81,575 (2.89)	81,475 (2.92)	100 (0.29)	
High school	859,126 (30.42)	857,181 (30.73)	1,945 (5.55)	
Bachelor or above	1,849,939 (65.50)	1,818,076 (65.18)	31,863 (90.99)	
Unknown	33,778 (1.20)	32,666 (1.17)	1,112 (3.18)	
Marital status, n (%)				< 0.001
Unmarried	789,496 (27.95)	788,170 (28.26)	1,326 (3.79)	
Married	1,677,120 (59.38)	1,648,773 (59.11)	28,347 (80.95)	
Unknown	357,802 (12.67)	352,455 (12.64)	5,347 (15.27)	
Age of father, years, Mean ± SD	31.95 ± 6.67	31.87 ± 6.64	38.13 ± 6.39	< 0.001
Race of father, n (%)				< 0.001
White	1,942,863 (68.79)	1,916,519 (68.71)	26,344 (75.23)	
Black	388,297 (13.75)	386,438 (13.85)	1,859 (5.31)	
Other	95,417 (3.38)	94,760 (3.40)	657 (1.88)	
Asian	174,795 (6.19)	170,426 (6.11)	4,369 (12.48)	
Unknown	223,046 (7.90)	221,255 (7.93)	1,791 (5.11)	
Educational level of father, n (%)				< 0.001
Less than high school	99,839 (3.53)	99,710 (3.57)	129 (0.37)	
High school	1,050,212 (37.18)	1,046,720 (37.52)	3,492 (9.97)	
Bachelor or above	1,602,222 (56.73)	1,572,117 (56.36)	30,105 (85.97)	
Unknown	72,145 (2.55)	70,851 (2.54)	1,294 (3.70)	
Smoking before pregnancy, n (%)				< 0.001
No	2,667,141 (94.43)	2,632,337 (94.37)	34,804 (99.38)	
Yes	157,277 (5.57)	157,061 (5.63)	216 (0.62)	
Smoking during pregnancy, n (%)				< 0.001
No	2,706,508 (95.83)	2,671,586 (95.78)	34,922 (99.72)	
Yes	117,910 (4.17)	117,812 (4.22)	98 (0.28)	
Total number of prenatal care visits for this pregnancy, number, M (Q_1_, Q_3_)	11.00 (9.00, 13.00)	11.00 (9.00, 13.00)	12.00 (10.00, 14.00)	< 0.001
Pre-pregnancy BMI, kg/m^2^, Mean ± SD	27.24 ± 6.57	27.25 ± 6.58	26.02 ± 5.70	< 0.001
Pre-pregnancy chronic diabetes, n (%)				0.023
No	2,799,766 (99.13)	2,765,091 (99.13)	34,675 (99.01)	
Yes	24,652 (0.87)	24,307 (0.87)	345 (0.99)	
Gestational diabetes, n (%)				< 0.001
No	2,603,840 (92.19)	2,572,908 (92.24)	30,932 (88.33)	
Yes	220,578 (7.81)	216,490 (7.76)	4,088 (11.67)	
Gestational hypertension, n (%)				< 0.001
No	2,590,390 (91.71)	2,559,677 (91.76)	30,713 (87.70)	
Yes	234,028 (8.29)	229,721 (8.24)	4,307 (12.30)	
Eclampsia, n (%)				0.100
No	2,817,716 (99.76)	2,782,794 (99.76)	34,922 (99.72)	
Yes	6,702 (0.24)	6,604 (0.24)	98 (0.28)	
PIH, n (%)				< 0.001
No	2584,830 (91.52)	2,554,194 (91.57)	30,636 (87.48)	
Yes	239,588 (8.48)	235,204 (8.43)	4,384 (12.52)	
Clinical chorioamnionitis or maternal fever during labor, n (%)				< 0.001
No	2,779,538 (98.41)	2,745,595 (98.43)	33,943 (96.92)	
Yes	44,880 (1.59)	43,803 (1.57)	1,077 (3.08)	
Previous preterm births, n (%)				0.224
No	2,733,365 (96.78)	2,699,434 (96.77)	33,931 (96.89)	
Yes	91,053 (3.22)	89,964 (3.23)	1,089 (3.11)	
Previous cesarean section, n (%)				0.481
No	2,389,278 (84.59)	2,359,606 (84.59)	29,672 (84.73)	
Yes	435,140 (15.41)	429,792 (15.41)	5,348 (15.27)	
Parity, n (%)				< 0.001
Primipara	1,095,213 (38.78)	1,075,044 (38.54)	20,169 (57.59)	
Multipara	1,729,205 (61.22)	1,714,354 (61.46)	14,851 (42.41)	
Gestational weight gain, pounds, M (Q_1_, Q_3_)	29.00 (20.00, 38.00)	29.00 (20.00, 38.00)	29.00 (21.00, 37.00)	0.415
Gestational age, weeks, Mean ± SD	38.75 ± 1.97	38.75 ± 1.96	38.54 ± 2.17	< 0.001
Adverse neonatal outcomes, n (%)				< 0.001
No	2,399,677 (84.96)	2,371,792 (85.03)	27,885 (79.63)	
Yes	424,741 (15.04)	417,606 (14.97)	7,135 (20.37)	
Neonatal weight, g, Mean ± SD	3,324.07 ± 521.64	3,324.12 ± 521.27	3,320.24 ± 549.90	0.189
Low birth weight, n (%)				< 0.001
No	2,672,002 (94.60)	2,639,294 (94.62)	32,708 (93.40)	
Yes	152,416 (5.40)	150,104 (5.38)	2,312 (6.60)	
Premature birth, n (%)				< 0.001
No	2,569,761 (90.98)	2,539,204 (91.03)	30,557 (87.26)	
Yes	254,657 (9.02)	250,194 (8.97)	4,463 (12.74)	
NICU admission, n (%)				< 0.001
No	2,621,722 (92.82)	2,590,534 (92.87)	31,188 (89.06)	
Yes	202,696 (7.18)	198,864 (7.13)	3,832 (10.94)	

Note: SC, spontaneous conception; ART, assisted reproductive technology; BMI, body mass index; PIH, pregnancy-induced hypertension; NICU, neonatal intensive care unit

All statistical analysis was two-sided and performed by SAS 9.4 software (SAS Institute Inc., Cary, NC, USA). Mediated effects analysis was performed using R 4.0.3 software (RMediation package). P-values less than 0.05 were considered significant.

## Results

### Participant characteristics

**Fig. 1:**
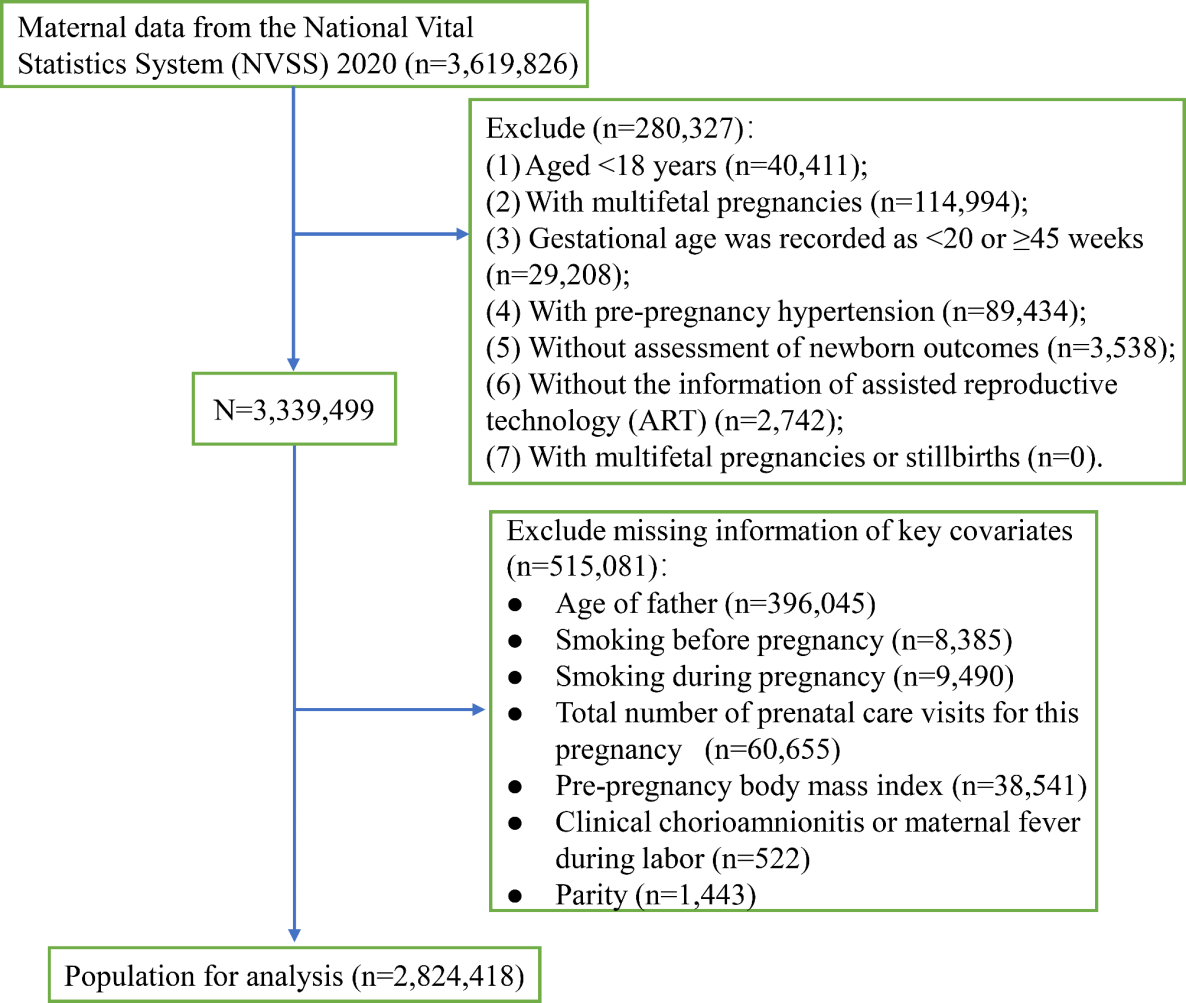
Participant flow char

In 2020, there were 3,619,826 pregnancies as recorded in the NVSS database. A total of 795,408 pregnancies were excluded, including 40,411 pregnancies aged < 18 years, 114,994 multifetal pregnancies, 29,208 pregnancies at < 20 or ≥ 45 weeks of gestational age, 89,434 pregnancies with pre-pregnancy hypertension, 3,538 pregnancies without assessment of newborn outcomes, 2,742 pregnancies without ART information, and 515,081 pregnancies with missing information on key covariates. After the screening, 2,824,418 pregnancies with singleton births were included in this study (Fig. 1). The maternal characteristics were shown in Table 1. Of these women, the mean age was 29.61 years, and 2,263,460 (80.14%) were younger than 35 years. There were 157,277 (5.57%) women who smoked before pregnancy and 117,910 (4.17%) women who smoked during pregnancy. The median pre-pregnancy BMI of women was 27.24 kg/m^2^ and the median birth weight of newborns was 3,324.07 g. There were 35,020 (1.24%) women who used ART, 239,588 (8.48%) women who had PIH, 91,053 (3.22%) women with previous preterm births, and 1,729,205 (61.22%) women with multipara. A total of 424,741 (15.04%) neonates had any adverse neonatal outcomes including 152,416 (5.40%) neonates with low birth weight, 254,657 (9.02%) neonates with premature birth, and 202,696 (7.18%) neonates admitted to the NICU. In addition, 128,158 (6.30%) pregnancies with pre-pregnancy BMI < 30 kg/m^2^ had PIH, while 111,430 (14.13%) pregnancies with pre-pregnancy BMI ≥ 30 kg/m^2^ had PIH.

### Effect of ART on PIH

Table 2 demonstrates the association between ART and PIH. Women who used ART were connected with a higher risk of PIH (OR = 1.55; 95%CI: 1.51–1.60). After adjusting for maternal age at delivery, race of mother, educational level of mother, marital status, age of father, race of father, educational level of father, smoking before pregnancy, smoking during pregnancy, total number of prenatal care visits for this pregnancy, pre-pregnancy BMI, pre-pregnancy chronic diabetes, gestational diabetes, clinical chorioamnionitis or maternal fever during labor, previous preterm births, previous cesarean section, parity, and gestational weight gain, the use of ART was still associated with an increased risk of PIH (OR = 1.42; 95%CI: 1.37–1.46). When stratified by aged (< 35 and ≥ 35 years) and parity (primipara and primipara), the use of ART was found to be connected with a higher risk of PIH in women < 35 years (OR = 1.43; 95%CI: 1.36–1.51), ≥ 35 years (OR = 1.50; 95%CI: 1.43–1.57), primipara (OR = 1.43; 95%CI: 1.37–1.49), and multipara (OR = 1.50; 95%CI: 1.41–1.58) compared with those who did not use ART.

**Table 2:** Effect of assisted reproductive technology (ART) on pregnancy-induced hypertension (PIH).

Populations	Variable	Crude Model		Adjusted Model	
		OR (95% CI)	*P*	OR (95% CI)	*P*
Total	ART	1.55 (1.51–1.60)	< 0.001	1.42 (1.37–1.46)	< 0.001
Age < 35 years	ART	1.54 (1.46–1.62)	< 0.001	1.43 (1.36–1.51)	< 0.001
Age ≥ 35 years	ART	1.50 (1.44–1.56)	< 0.001	1.50 (1.43–1.57)	< 0.001
Primipara	ART	1.37 (1.32–1.42)	< 0.001	1.43 (1.37–1.49)	< 0.001
Multipara	ART	1.50 (1.42–1.58)	< 0.001	1.50 (1.41–1.58)	< 0.001

Note: OR, odds ratio; 95%CI, 95% confidence interval; crude model, univariate logistic regression model; adjusted model, multivariate logistic regression model adjusted for maternal age at delivery (not in age subgroups), race of mother, educational level of mother, marital status, age of father, race of father, educational level of father, smoking before pregnancy, smoking during pregnancy, total number of prenatal care visits for this pregnancy, pre-pregnancy BMI, pre-pregnancy chronic diabetes, gestational diabetes, clinical chorioamnionitis or maternal fever during labor, previous preterm births, previous cesarean section, parity (not in parity subgroups), and gestational weight gain

### Mediation effect of PIH in the association between ART and adverse neonatal outcomes

**Fig. 2:**
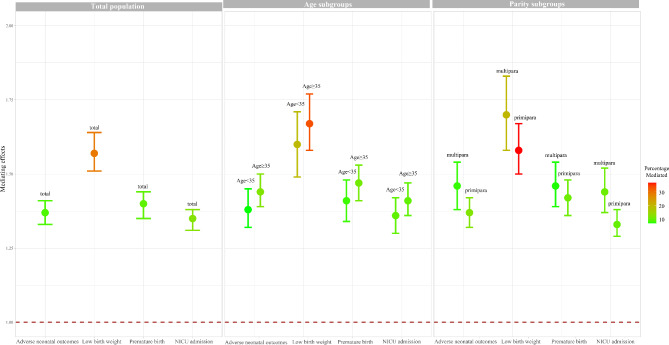
Mediation effect of pregnancy-induced hypertension (PIH) in the relationship between assisted reproductive technology (ART) and adverse neonatal outcomes. NICU, neonatal intensive care unit

Table 3; Fig. 2 show the mediation effect of PIH in the relationship between ART and adverse neonatal outcomes. Women who used ART were associated with a higher risk of any adverse neonatal outcomes (OR = 1.45; 95%CI: 1.42–1.49). After adjusting for all confounders (model 2), the use of ART was connected with a higher risk of any adverse neonatal outcomes (OR = 1.47; 95%CI: 1.43–1.51). After further adjusting for PIH (model 3), the use of ART was still associated with an increased risk of any adverse neonatal outcomes (OR = 1.43; 95%CI: 1.39–1.47). The distribution-of-the-product was 0.31 (95%CI: 0.28–0.34), and 8.51% of the association between ART and any adverse neonatal outcomes was mediated through PIH. When stratified by aged and parity, the distribution-of-the-product was 0.32 (95%CI: 0.28–0.37) for women aged < 35 years, 0.37 (95%CI: 0.33–0.41) for women aged ≥ 35 years, 0.31 (95%CI: 0.28–0.35) for women with primipara, and 0.38 (95%CI: 0.32–0.43) for women with multipara. In different adverse neonatal outcomes: the distribution-of-the-product was 0.45 (95%CI: 0.41–0.50) when neonatal low birth weight was the outcome, and 29.17% of the association between ART and neonatal low birth weight was mediated through PIH; the distribution-of-the-product was 0.33 (95%CI: 0.30–0.37) when neonatal premature birth was the outcome, and 9.37% of the association between ART and neonatal premature birth was mediated through PIH; and the distribution-of-the-product was 0.30 (95%CI: 0.27–0.33) when NICU admission was the outcome, and 12.20% of the association between ART and NICU admission was mediated through PIH.

**Table 3:** Mediation effect of pregnancy-induced hypertension (PIH) in the relationship between assisted reproductive technology (ART) and adverse neonatal outcomes

Outcomes	Populations	Model 1	Model 2	Model 3	Distribution-of-the-product (95% CI)	Indirect Effect (95% CI)	Percentage Mediated (%)
		OR (95% *CI*)	OR1 (95% CI)	OR2 (95% CI)			
Adverse neonatal outcomes	Total	1.45 (1.42–1.49)	1.47 (1.43–1.51)	1.43 (1.39–1.47)	0.31 (0.28–0.34)	1.37 (1.33–1.41)	8.51
	Age < 35 years	1.43 (1.37–1.49)	1.59 (1.52–1.66)	1.55 (1.48–1.61)	0.32 (0.28–0.37)	1.38 (1.32–1.45)	6.78
	Age ≥ 35 years	1.34 (1.29–1.39)	1.42 (1.37–1.47)	1.37 (1.33–1.42)	0.37 (0.33–0.41)	1.44 (1.39–1.50)	11.90
	Primipara	1.41 (1.37–1.46)	1.42 (1.37–1.47)	1.37 (1.32–1.42)	0.31 (0.28–0.35)	1.37 (1.32–1.42)	11.90
	Multipara	1.41 (1.36–1.47)	1.53 (1.47–1.60)	1.49 (1.43–1.56)	0.38 (0.32–0.43)	1.46 (1.38–1.54)	7.55
Low birth weight	Total	1.24 (1.19–1.30)	1.24 (1.19–1.30)	1.17 (1.12–1.23)	0.45 (0.41–0.50)	1.57 (1.51–1.64)	29.17
	Age < 35 years	1.20 (1.12–1.29)	1.36 (1.26–1.45)	1.29 (1.20–1.38)	0.47 (0.40–0.54)	1.60 (1.49–1.71)	19.44
	Age ≥ 35 years	1.20 (1.13–1.27)	1.21 (1.14–1.28)	1.14 (1.08–1.21)	0.51 (0.46–0.57)	1.67 (1.58–1.77)	33.33
	Primipara	1.17 (1.11–1.23)	1.19 (1.13–1.26)	1.12 (1.06–1.19)	0.46 (0.41–0.51)	1.58 (1.50–1.67)	36.84
	Multipara	1.15 (1.07–1.24)	1.30 (1.21–1.40)	1.24 (1.15–1.34)	0.53 (0.46–0.60)	1.70 (1.58–1.83)	20.00
Premature birth	Total	1.48 (1.44–1.53)	1.64 (1.58–1.69)	1.58 (1.53–1.64)	0.33 (0.30–0.37)	1.40 (1.35–1.44)	9.37
	Age < 35 years	1.41 (1.34–1.49)	1.77 (1.68–1.86)	1.71 (1.62–1.81)	0.35 (0.30–0.40)	1.41 (1.34–1.48)	7.79
	Age ≥ 35 years	1.34 (1.29–1.39)	1.56 (1.50–1.63)	1.50 (1.44–1.57)	0.38 (0.34–0.43)	1.47 (1.41–1.53)	10.71
	Primipara	1.50 (1.43–1.56)	1.58 (1.51–1.65)	1.52 (1.45–1.59)	0.35 (0.31–0.39)	1.42 (1.36–1.48)	10.34
	Multipara	1.52 (1.45–1.59)	1.71 (1.63–1.79)	1.66 (1.58–1.74)	0.38 (0.33–0.43)	1.46 (1.39–1.54)	7.04
NICU admission	Total	1.60 (1.55–1.66)	1.41 (1.36–1.46)	1.36 (1.32–1.41)	0.30 (0.27–0.33)	1.35 (1.31–1.38)	12.20
	Age < 35 years	1.60 (1.51–1.68)	1.53 (1.44–1.61)	1.48 (1.40–1.56)	0.30 (0.26–0.35)	1.36 (1.30–1.42)	9.43
	Age ≥ 35 years	1.44 (1.38–1.50)	1.42 (1.36–1.49)	1.38 (1.31–1.44)	0.35 (0.31–0.38)	1.41 (1.36–1.47)	9.52
	Primipara	1.53 (1.47–1.60)	1.41 (1.35–1.48)	1.37 (1.31–1.43)	0.29 (0.25–0.32)	1.33 (1.29–1.38)	9.76
	Multipara	1.48 (1.40–1.57)	1.39 (1.32–1.48)	1.35 (1.28–1.43)	0.37 (0.31–0.42)	1.44 (1.37–1.52)	10.26

Note: OR, odds ratio; 95%CI, 95% confidence interval; model 1, univariate logistic regression model; model 2, multivariate logistic regression model adjusted for maternal age at delivery (not in age subgroups), race of mother, educational level of mother, marital status, age of father, race of father, educational level of father, smoking before pregnancy, smoking during pregnancy, total number of prenatal care visits for this pregnancy, pre-pregnancy BMI, pre-pregnancy chronic diabetes, gestational diabetes, clinical chorioamnionitis or maternal fever during labor, previous preterm births, previous cesarean section, parity (not in parity subgroups), and gestational weight gain; model 3 further adjusted for PIH (intermediate variable) based on model 2; NICU, neonatal intensive care unit

*Percentage mediated: The percentages of mediated explained by PIH were calculated using the formula (OR1 - OR2) / (OR1–1) * 100

In addition, interaction analysis of the effect of ART and PIH on adverse neonatal outcomes was performed (Supplement Table 5). The results demonstrated that no interaction effect between ART and PIH was found for adverse neonatal outcomes.

## Discussion

The main finding of this study was that PIH may play a mediating effect in the association between ART and adverse neonatal outcomes. Women who used ART were independently associated with a higher risk of PIH and adverse neonatal outcomes. An 8.51% association between ART and adverse neonatal outcomes was mediated through PIH. The mediating effects of PIH on the association between ART and adverse neonatal outcomes were observed in women aged < 35 years, aged ≥ 35 years, primipara, and multipara.

ART has been reported to be associated with a higher risk of adverse maternal and neonatal outcomes such as cesarean delivery, preterm birth, and low birth weight [10, 11]. Our results demonstrated that ART was connected with a higher risk of adverse neonatal outcomes, including premature birth, low birth weight, and admission to NICU. However, the potential mechanisms underlying the effects of ART on adverse neonatal outcomes remain uncertain. A systematic review by Kawwass et al. summarized the possible mechanisms including ovarian stimulation, uterine hormonal environment, gamete manipulation, and their underlying infertility [26]. Several studies have found that the in vitro manipulation process of ART may cause abnormal DNA methylation in human gametes, embryos, placentas, and umbilical cord samples [8, 9]. Treatments and procedures during ART may also affect gamete development and increase the susceptibility of children to diseases [27]. Many ART parameters have been reported that may adversely affect implantation or placentation [28, 29]. Inadequate trophoblast invasion at implantation, particularly restricted intravascular invasion has been implicated in the pathophysiology of preeclampsia, premature rupture of membranes, preterm birth, and intrauterine growth restriction resulting in small infants [30]. Several studies have confirmed that abnormal placentation occurs due to reduced trophoblast invasion of the meconium and uterine spiral arteries and abnormal cell survival and apoptosis [31, 32]. Excessive invasion of the trophoblast is related to placental hyperplasia across the metaphase or inadequate metaphase formation [33]. In addition, the pregnant women’s physiological conditions can also affect adverse neonatal outcomes. Pregnancies in infertile women with or without infertility treatment have been reported to be associated with more complications, lower birth weights, and shorter gestations [34].

A systematic review and meta-analysis indicated that both ART singleton pregnancies and multiple pregnancies were linked to higher odds of PIH compared to spontaneous conception [14]. Our results also supported that ART was related to a higher risk of PIH. However, the underlying mechanisms between ART and PIH are not well understood [10, 35]. This may be related to abnormal placentation, epigenetic effects, and underlying infertility [16, 36]. PIH is one of the leading causes of adverse maternal and neonatal outcomes during pregnancy [16, 37]. In singleton pregnancies, the occurrence of PIH predisposes the infant to adverse neonatal outcomes including preterm birth, low birth weight, and small for gestational age [17, 18]. The pathophysiological mechanisms of PIH development remain unclear and may be related to fetal-maternal immunity [38–40]. PIH may arise from poor maternal immune tolerance and abnormal interactions between immune cells, trophoblast cells, and metaphase stromal cells after embryo implantation [39, 40]. Our results found that PIH may play a mediating effect in the association between ART and adverse neonatal outcomes (premature birth, low birth weight, and admission to NICU). In addition, we ruled out a possible interaction between ART and PIH by interaction analysis, confirming that the mediating effect of PIH between ART and adverse neonatal outcomes is reliable. Stern et al. found that both placental abnormalities and PIH mediated the association between ART and preterm birth [20]. Compared with the previous study, our study identified mediating effects of PIH in the association between ART and different adverse neonatal outcomes. Our results may provide more evidence for the association between ART and adverse neonatal outcomes. In addition, the clinical implication of the mediating role of PIH between ART and adverse neonatal outcomes suggests that the timely use of interventions to reduce the risk of PIH in women after ART can reduce the impact of ART on adverse neonatal outcomes.

The results of this study, based on a large sample data from the NVSS database, provided strong evidence for the association between ART, PIH, and adverse neonatal outcomes. This study performed subgroup analysis according to maternal age (< 35 years and ≥ 35 years) and parity (primipara and multipara), which allowed the results to be applied to a more specific population. However, several limitations should be considered. First, specific ART types (e.g., conventional in vitro fertilization and intracytoplasmic sperm injection) were not available in the NVSS database, and there is necessary to further investigate neonatal outcomes under different ART types and the role of PIH in them to make the formulation of clinical strategies more targeted. Second, some possible confounders such as dietary patterns during pregnancy, treatments, and alcohol and drug use during pregnancy were not available due to the limitations of data collection in the NVSS database. Third, we included only women with singleton pregnancies, and the application of the findings in women with multiple pregnancies needs further study.

## Conclusions

In this large-scale population-based study, we found that PIH may play a mediating effect in the association between ART and adverse neonatal outcomes (premature birth, low birth weight, and admission to NICU). The mediating effect of PIH between ART and adverse neonatal outcomes were found in the woman of different ages (< 35 years and ≥ 35 years) and parities (primipara and multipara). Further studies are needed to determine the mechanisms by which AR affects PIH so that interventions to reduce PIH can be developed to reduce adverse neonatal outcomes associated with ART.

## Electronic supplementary material

Below is the link to the electronic supplementary material.

**Additional file 1:** Supplementary Tables

## Data Availability

The datasets generated and/or analyzed during the current study are available in the NVSS database, https://www.cdc.gov/nchs/nvss/index.htm.

## Abbreviations

ART: Assisted reproductive technology
PIH: Pregnancy-induced hypertension
NVSS: National Vital Statistics System
NCHS: National Center for Health Statistics
CDC: Centers for Disease Control and Prevention
BMI: Body mass index
NICU: Neonatal intensive care unit
SD: Standard deviation
OR: Odds ratio
CI: Confidence interval
