# Supplementary material for: Mediation effect of pregnancy-induced hypertension on the association between assisted reproductive technology and adverse neonatal outcomes: a population-based study
Source: BMC Pregnancy Childbirth. 2023 May 25;23:385. doi: 10.1186/s12884-023-05694-3 (PMC10210490; doi:10.1186/s12884-023-05694-3)
Supplement: Supplementary file 1 — Additional file 1: Supplementary Tables [file 12884_2023_5694_MOESM1_ESM.docx]

**Supplement Table 1.** Univariate logistic regression analysis for confounders screening when any adverse neonatal outcomes was the outcome variable.

| **Variables** | **β** | **S.E** | **OR (95%CI)** | ***P*** |
| --- | --- | --- | --- | --- |
| ART |  |  |  |  |
| No |  |  | Ref |  |
| Yes | 0.37 | 0.013 | 1.45 (1.42-1.49) | <0.001 |
| Pregnancy-induced hypertension |  |  |  |  |
| No |  |  | Ref |  |
| Yes | 0.95 | 0.005 | 2.58 (2.55-2.60) | <0.001 |
| Maternal age at delivery | 0.00 | 0.000 | 1.01 (1.01-1.01) | <0.001 |
| Age subgroups |  |  |  |  |
| <35 years |  |  | Ref |  |
| ≥35years | 0.15 | 0.004 | 1.16 (1.15-1.17) | <0.001 |
| Race of mother |  |  |  |  |
| White |  |  | Ref |  |
| Black | 0.47 | 0.005 | 1.61 (1.59-1.62) | <0.001 |
| Other | 0.16 | 0.009 | 1.18 (1.16-1.20) | <0.001 |
| Asian | 0.11 | 0.006 | 1.12 (1.10-1.13) | <0.001 |
| Educational level of mother |  |  |  |  |
| Less than high school |  |  | Ref |  |
| High school | 0.00 | 0.010 | 1.01 (0.98-1.02) | 0.855 |
| Bachelor or above | -0.27 | 0.009 | 0.76 (0.75-0.78) | <0.001 |
| Unknown | -0.14 | 0.018 | 0.87 (0.84-0.90) | <0.001 |
| Marriage |  |  |  |  |
| Unmarried |  |  | Ref |  |
| Married | -0.33 | 0.004 | 0.72 (0.71-0.72) | <0.001 |
| Unknown | -0.40 | 0.006 | 0.67 (0.66-0.68) | <0.001 |
| Age of father | 0.00 | 0.000 | 1.01 (1.01-1.01) | <0.001 |
| Race of father |  |  |  |  |
| White |  |  | Ref |  |
| Black | 0.46 | 0.005 | 1.58 (1.57-1.60) | <0.001 |
| Other | 0.14 | 0.009 | 1.15 (1.13-1.17) | <0.001 |
| Asian | 0.11 | 0.007 | 1.12 (1.11-1.14) | <0.001 |
| Unknown | 0.25 | 0.006 | 1.29 (1.27-1.30) | <0.001 |
| Educational level of father |  |  |  |  |
| Less than high school |  |  | Ref |  |
| High school | 0.01 | 0.009 | 1.01 (1.01-1.03) | 0.137 |
| Bachelor or above | -0.27 | 0.009 | 0.76 (0.75-0.77) | <0.001 |
| Unknown | 0.08 | 0.013 | 1.08 (1.05-1.11) | <0.001 |
| Smoking before pregnancy | 0.02 | 0.000 | 1.02 (1.02-1.02) | <0.001 |
| Smoking during pregnancy | 0.01 | 0.000 | 1.01 (1.01-1.01) | <0.001 |
| Total number of prenatal care visits for this pregnancy | -0.07 | 0.000 | 0.93 (0.93-0.93) | <0.001 |
| Pre-pregnancy BMI | 0.01 | 0.000 | 1.01 (1.01-1.01) | <0.001 |
| Pre-pregnancy chronic diabetes |  |  |  |  |
| No |  |  | Ref |  |
| Yes | 1.14 | 0.013 | 3.14 (3.06-3.22) | <0.001 |
| Gestational diabetes |  |  |  |  |
| No |  |  | Ref |  |
| Yes | 0.37 | 0.006 | 1.44 (1.43-1.46) | <0.001 |
| Clinical chorioamnionitis or maternal fever during labor |  |  |  |  |
| No |  |  | Ref |  |
| Yes | 0.67 | 0.011 | 1.95 (1.91-1.99) | <0.001 |
| Previous preterm births |  |  |  |  |
| No |  |  | Ref |  |
| Yes | 1.05 | 0.007 | 2.85 (2.81-2.89) | <0.001 |
| Previous cesarean section |  |  |  |  |
| No |  |  | Ref |  |
| Yes | 0.25 | 0.004 | 1.29 (1.28-1.30) | <0.001 |
| Parity |  |  |  |  |
| Primipara |  |  | Ref |  |
| Multipara | -0.15 | 0.003 | 0.86 (0.86-0.87) | <0.001 |
| Gestational weight gain | -0.01 | 0.000 | 0.99 (0.99-0.99) | <0.001 |

Note: ART, assisted reproductive technology, BMI, body mass index; OR, odds ratio; 95%CI, 95% confidence interval; S.E, standard error; Ref, reference.

**Supplement Table 2.** Univariate logistic regression analysis for confounders screening when low birth weight was the outcome variable.

| **Variables** | **β** | **S.E** | **OR (95%CI)** | ***P*** |
| --- | --- | --- | --- | --- |
| ART |  |  |  |  |
| No |  |  | Ref |  |
| Yes | 0.22 | 0.022 | 1.24 (1.19-1.30) | <0.001 |
| Pregnancy-induced hypertension |  |  |  |  |
| No |  |  | Ref |  |
| Yes | 1.19 | 0.007 | 3.30 (3.26-3.34) | <0.001 |
| Maternal age at delivery | -0.01 | 0.000 | 0.99 (0.99-0.99) | <0.001 |
| Age subgroups |  |  |  |  |
| <35 years |  |  | Ref |  |
| ≥35years | 0.09 | 0.006 | 1.10 (1.08-1.11) | <0.001 |
| Race of mother |  |  |  |  |
| White |  |  | Ref |  |
| Black | 0.73 | 0.007 | 2.07 (2.04-2.10) | <0.001 |
| Other | 0.20 | 0.014 | 1.22 (1.19-1.26) | <0.001 |
| Asian | 0.39 | 0.009 | 1.47 (1.44-1.50) | <0.001 |
| Educational level of mother |  |  |  |  |
| Less than high school |  |  | Ref |  |
| High school | 0.23 | 0.016 | 1.26 (1.22-1.30) | <0.001 |
| Bachelor or above | -0.09 | 0.016 | 0.91 (0.89-0.94) | <0.001 |
| Unknown | 0.09 | 0.028 | 1.09 (1.03-1.16) | 0.002 |
| Marriage |  |  |  |  |
| Unmarried |  |  | Ref |  |
| Married | -0.46 | 0.006 | 0.63 (0.63-0.64) | <0.001 |
| Unknown | -0.42 | 0.009 | 0.66 (0.64-0.67) | <0.001 |
| Age of father | -0.00 | 0.000 | 0.99 (0.99-0.99) | <0.001 |
| Race of father |  |  |  |  |
| White |  |  | Ref |  |
| Black | 0.70 | 0.007 | 2.01 (1.98-2.03) | <0.001 |
| Other | 0.23 | 0.014 | 1.26 (1.22-1.29) | <0.001 |
| Asian | 0.43 | 0.010 | 1.54 (1.51-1.57) | <0.001 |
| Unknown | 0.27 | 0.010 | 1.31 (1.28-1.33) | <0.001 |
| Educational level of father |  |  |  |  |
| Less than high school |  |  | Ref |  |
| High school | 0.20 | 0.015 | 1.23 (1.19-1.26) | <0.001 |
| Bachelor or above | -0.14 | 0.015 | 0.87 (0.84-0.89) | <0.001 |
| Unknown | 0.25 | 0.020 | 1.29 (1.24-1.34) | <0.001 |
| Smoking before pregnancy | 0.03 | 0.000 | 1.03 (1.03-1.03) | <0.001 |
| Smoking during pregnancy | 0.02 | 0.000 | 1.02 (1.01-1.02) | <0.001 |
| Total number of prenatal care visits for this pregnancy | -0.11 | 0.001 | 0.90 (0.90-0.90) | <0.001 |
| Pre-pregnancy BMI | -0.00 | 0.000 | 0.99 (0.99-0.99) | <0.001 |
| Pre-pregnancy chronic diabetes |  |  |  |  |
| No |  |  | Ref |  |
| Yes | 0.54 | 0.023 | 1.72 (1.65-1.80) | <0.001 |
| Gestational diabetes |  |  |  |  |
| No |  |  | Ref |  |
| Yes | 0.15 | 0.009 | 1.16 (1.14-1.18) | <0.001 |
| Clinical chorioamnionitis or maternal fever during labor |  |  |  |  |
| No |  |  | Ref |  |
| Yes | -0.33 | 0.024 | 0.72 (0.69-0.76) | <0.001 |
| Previous preterm births |  |  |  |  |
| No |  |  | Ref |  |
| Yes | 1.15 | 0.010 | 3.14 (3.08-3.20) | <0.001 |
| Previous cesarean section |  |  |  |  |
| No |  |  | Ref |  |
| Yes | 0.04 | 0.007 | 1.04 (1.03-1.06) | <0.001 |
| Parity |  |  |  |  |
| Primipara |  |  | Ref |  |
| Multipara | -0.35 | 0.005 | 0.70 (0.70-0.71) | <0.001 |
| Gestational weight gain | -0.02 | 0.000 | 0.98 (0.98-0.98) | <0.001 |

Note: ART, assisted reproductive technology, BMI, body mass index; OR, odds ratio; 95%CI, 95% confidence interval; S.E, standard error; Ref, reference.

**Supplement Table 3.** Univariate logistic regression analysis for confounders screening when premature birth was the outcome variable.

| **Variables** | **β** | **S.E** | **OR (95%CI)** | ***P*** |
| --- | --- | --- | --- | --- |
| ART |  |  |  |  |
| No |  |  | Ref |  |
| Yes | 0.39 | 0.016 | 1.48 (1.44-1.53) | <0.001 |
| Pregnancy-induced hypertension |  |  |  |  |
| No |  |  | Ref |  |
| Yes | 0.97 | 0.006 | 2.64 (2.61-2.67) | <0.001 |
| Maternal age at delivery | 0.01 | 0.000 | 1.01 (1.01-1.01) | <0.001 |
| Age subgroups |  |  |  |  |
| <35 years |  |  | Ref |  |
| ≥35years | 0.21 | 0.005 | 1.24 (1.22-1.25) | <0.001 |
| Race of mother |  |  |  |  |
| White |  |  | Ref |  |
| Black | 0.45 | 0.006 | 1.57 (1.56-1.59) | <0.001 |
| Other | 0.19 | 0.011 | 1.21 (1.18-1.23) | <0.001 |
| Asian | 0.01 | 0.008 | 1.01 (0.99-1.02) | 0.582 |
| Educational level of mother |  |  |  |  |
| Less than high school |  |  | Ref |  |
| High school | -0.12 | 0.011 | 0.88 (0.86-0.90) | <0.001 |
| Bachelor or above | -0.45 | 0.011 | 0.64 (0.63-0.65) | <0.001 |
| Unknown | -0.25 | 0.021 | 0.78 (0.75-0.81) | <0.001 |
| Marriage |  |  |  |  |
| Unmarried |  |  | Ref |  |
| Married | -0.34 | 0.005 | 0.71 (0.71-0.72) | <0.001 |
| Unknown | -0.37 | 0.007 | 0.69 (0.68-0.70) | <0.001 |
| Age of father | 0.01 | 0.000 | 1.01 (1.01-1.01) | <0.001 |
| Race of father |  |  |  |  |
| White |  |  | Ref |  |
| Black | 0.44 | 0.006 | 1.55 (1.54-1.57) | <0.001 |
| Other | 0.16 | 0.011 | 1.17 (1.15-1.20) | <0.001 |
| Asian | -0.03 | 0.009 | 0.97 (0.95-0.99) | 0.001 |
| Unknown | 0.31 | 0.007 | 1.36 (1.34-1.38) | <0.001 |
| Educational level of father |  |  |  |  |
| Less than high school |  |  | Ref |  |
| High school | -0.10 | 0.010 | 0.90 (0.88-0.92) | <0.001 |
| Bachelor or above | -0.44 | 0.010 | 0.64 (0.63-0.66) | <0.001 |
| Unknown | -0.02 | 0.015 | 0.98 (0.96-1.01) | 0.314 |
| Smoking before pregnancy | 0.02 | 0.000 | 1.02 (1.02-1.02) | <0.001 |
| Smoking during pregnancy | 0.01 | 0.000 | 1.01 (1.01-1.01) | <0.001 |
| Total number of prenatal care visits for this pregnancy | -0.11 | 0.001 | 0.90 (0.90-0.90) | <0.001 |
| Pre-pregnancy BMI | 0.02 | 0.000 | 1.02 (1.01-1.02) | <0.001 |
| Pre-pregnancy chronic diabetes |  |  |  |  |
| No |  |  | Ref |  |
| Yes | 1.04 | 0.016 | 2.84 (2.75-2.93) | <0.001 |
| Gestational diabetes |  |  |  |  |
| No |  |  | Ref |  |
| Yes | 0.35 | 0.007 | 1.42 (1.40-1.44) | <0.001 |
| Clinical chorioamnionitis or maternal fever during labor |  |  |  |  |
| No |  |  | Ref |  |
| Yes | -0.35 | 0.019 | 0.71 (0.68-0.73) | <0.001 |
| Previous preterm births |  |  |  |  |
| No |  |  | Ref |  |
| Yes | 1.24 | 0.008 | 3.45 (3.40-3.51) | <0.001 |
| Previous cesarean section |  |  |  |  |
| No |  |  | Ref |  |
| Yes | 0.30 | 0.005 | 1.34 (1.33-1.36) | <0.001 |
| Parity |  |  |  |  |
| Primipara |  |  | Ref |  |
| Multipara | 0.08 | 0.004 | 1.08 (1.07-1.09) | <0.001 |
| Gestational weight gain | -0.01 | 0.000 | 0.99 (0.99-0.99) | <0.001 |

Note: ART, assisted reproductive technology, BMI, body mass index; OR, odds ratio; 95%CI, 95% confidence interval; S.E, standard error; Ref, reference.

**Supplement Table 4.** Univariate logistic regression analysis for confounders screening when NICU admission was the outcome variable.

| **Variables** | **β** | **S.E** | **OR (95%CI)** | ***P*** |
| --- | --- | --- | --- | --- |
| ART |  |  |  |  |
| No |  |  | Ref |  |
| Yes | 0.47 | 0.017 | 1.60 (1.55-1.66) | <0.001 |
| Pregnancy-induced hypertension |  |  |  |  |
| No |  |  | Ref |  |
| Yes | 1.01 | 0.006 | 2.74 (2.70-2.77) | <0.001 |
| Maternal age at delivery | 0.01 | 0.000 | 1.01 (1.01-1.01) | <0.001 |
| Age subgroups |  |  |  |  |
| <35 years |  |  | Ref |  |
| ≥35years | 0.18 | 0.006 | 1.19 (1.18-1.21) | <0.001 |
| Race of mother |  |  |  |  |
| White |  |  | Ref |  |
| Black | 0.33 | 0.006 | 1.40 (1.38-1.41) | <0.001 |
| Other | 0.08 | 0.012 | 1.09 (1.06-1.11) | <0.001 |
| Asian | 0.02 | 0.009 | 1.02 (1.01-1.04) | 0.046 |
| Educational level of mother |  |  |  |  |
| Less than high school |  |  | Ref |  |
| High school | 0.15 | 0.015 | 1.16 (1.13-1.19) | <0.001 |
| Bachelor or above | 0.04 | 0.014 | 1.04 (1.01-1.07) | 0.010 |
| Unknown | 0.12 | 0.025 | 1.12 (1.07-1.18) | <0.001 |
| Marriage |  |  |  |  |
| Unmarried |  |  | Ref |  |
| Married | -0.19 | 0.005 | 0.83 (0.82-0.84) | <0.001 |
| Unknown | -0.32 | 0.008 | 0.73 (0.72-0.74) | <0.001 |
| Age of father | 0.01 | 0.000 | 1.01 (1.01-1.01) | <0.001 |
| Race of father |  |  |  |  |
| White |  |  | Ref |  |
| Black | 0.31 | 0.006 | 1.37 (1.35-1.38) | <0.001 |
| Other | 0.03 | 0.013 | 1.03 (1.01-1.06) | 0.012 |
| Asian | -0.01 | 0.010 | 0.99 (0.97-1.01) | 0.390 |
| Unknown | 0.12 | 0.008 | 1.13 (1.11-1.15) | <0.001 |
| Educational level of father |  |  |  |  |
| Less than high school |  |  | Ref |  |
| High school | 0.16 | 0.013 | 1.17 (1.14-1.20) | <0.001 |
| Bachelor or above | 0.00 | 0.013 | 1.01 (0.98-1.03) | 0.916 |
| Unknown | 0.24 | 0.018 | 1.27 (1.23-1.32) | <0.001 |
| Smoking before pregnancy | 0.01 | 0.000 | 1.01 (1.01-1.01) | <0.001 |
| Smoking during pregnancy | 0.01 | 0.000 | 1.01 (1.01-1.01) | <0.001 |
| Total number of prenatal care visits for this pregnancy | -0.06 | 0.001 | 0.94 (0.94-0.94) | <0.001 |
| Pre-pregnancy BMI | 0.02 | 0.000 | 1.02 (1.02-1.02) | <0.001 |
| Pre-pregnancy chronic diabetes |  |  |  |  |
| No |  |  | Ref |  |
| Yes | 1.45 | 0.015 | 4.28 (4.16-4.41) | <0.001 |
| Gestational diabetes |  |  |  |  |
| No |  |  | Ref |  |
| Yes | 0.46 | 0.007 | 1.59 (1.56-1.61) | <0.001 |
| Clinical chorioamnionitis or maternal fever during labor |  |  |  |  |
| No |  |  | Ref |  |
| Yes | 1.30 | 0.012 | 3.65 (3.57-3.74) | <0.001 |
| Previous preterm births |  |  |  |  |
| No |  |  | Ref |  |
| Yes | 0.98 | 0.009 | 2.65 (2.60-2.70) | <0.001 |
| Previous cesarean section |  |  |  |  |
| No |  |  | Ref |  |
| Yes | 0.37 | 0.006 | 1.44 (1.42-1.46) | <0.001 |
| Parity |  |  |  |  |
| Primipara |  |  | Ref |  |
| Multipara | -0.31 | 0.005 | 0.74 (0.73-0.74) | <0.001 |
| Gestational weight gain | -0.01 | 0.000 | 0.99 (0.99-0.99) | <0.001 |

Note: ART, assisted reproductive technology, BMI, body mass index; OR, odds ratio; 95%CI, 95% confidence interval; S.E, standard error; Ref, reference; NICU, neonatal intensive care unit.

**Supplement Table 5.** Interaction analysis of the effects of assisted reproductive technology (ART) and pregnancy-induced hypertension (PIH) on adverse neonatal outcomes.

| **Outcomes and populations** | **Variables** | **Crude Model** | | **Adjusted Model** | |
| --- | --- | --- | --- | --- | --- |
|  |  | **OR (95% CI*)*** | ***P*** | **OR (95% CI)** | ***P*** |
| **Adverse neonatal outcomes** |  |  |  |  |  |
| Total sample | ART | 1.40 (1.36-1.44) | <0.001 | 1.43 (1.39-1.47) | <0.001 |
|  | PIH | 2.57 (2.55-2.60) | <0.001 | 2.47 (2.44-2.49) | <0.001 |
|  | ART *PIH | 0.97 (0.90-1.03) | 0.320 | 0.99 (0.93-1.07) | 0.842 |
| Age<35 years | ART | 1.39 (1.32-1.45) | <0.001 | 1.55 (1.48-1.63) | <0.001 |
|  | PIH | 2.52 (2.49-2.54) | <0.001 | 2.46 (2.44-2.49) | <0.001 |
|  | ART *PIH | 0.95 (0.85-1.06) | 0.344 | 0.98 (0.88-1.10) | 0.728 |
| Age≥35 years | ART | 1.30 (1.26-1.35) | <0.001 | 1.38 (1.33-1.43) | <0.001 |
|  | PIH | 2.77 (2.71-2.83) | <0.001 | 2.48 (2.43-2.54) | <0.001 |
|  | ART *PIH | 0.92 (0.84-1.01) | 0.065 | 0.98 (0.90-1.08) | 0.687 |
| Primipara | ART | 1.38 (1.33-1.43) | <0.001 | 1.38 (1.32-1.43) | <0.001 |
|  | PIH | 2.38 (2.35-2.41) | <0.001 | 2.39 (2.35-2.42) | <0.001 |
|  | ART *PIH | 0.98 (0.90-1.07) | 0.618 | 0.99 (0.91-1.08) | 0.838 |
| Multipara | ART | 1.37 (1.31-1.43) | <0.001 | 1.48 (1.42-1.55) | <0.001 |
|  | PIH | 2.71 (2.67-2.74) | <0.001 | 2.55 (2.51-2.58) | <0.001 |
|  | ART *PIH | 1.01 (0.90-1.13) | 0.901 | 1.04 (0.92-1.17) | 0.519 |
| **Low birth weight** |  |  |  |  |  |
| Total sample | ART | 1.14 (1.08-1.19) | <0.001 | 1.15 (1.09-1.21) | <0.001 |
|  | PIH | 3.29 (3.25-3.33) | <0.001 | 3.66 (3.61-3.71) | <0.001 |
|  | ART *PIH | 1.08 (0.98-1.19) | 0.105 | 1.08 (0.98-1.19) | 0.135 |
| Age<35 years | ART | 1.11 (1.03-1.21) | 0.009 | 1.27 (1.17-1.37) | <0.001 |
|  | PIH | 3.24 (3.19-3.29) | <0.001 | 3.70 (3.65-3.76) | <0.001 |
|  | ART*PIH | 1.04 (0.89-1.21) | 0.608 | 1.06 (0.91-1.25) | 0.444 |
| Age≥35 years | ART | 1.10 (1.03-1.18) | 0.004 | 1.11 (1.04-1.19) | 0.003 |
|  | PIH | 3.48 (3.38-3.58) | <0.001 | 3.54 (3.43-3.65) | <0.001 |
|  | ART*PIH | 1.06 (0.94-1.20) | 0.369 | 1.11 (0.97-1.26) | 0.119 |
| Primipara | ART | 1.08 (1.02-1.16) | 0.013 | 1.10 (1.03-1.18) | 0.003 |
|  | PIH | 3.05 (2.99-3.10) | <0.001 | 3.59 (3.52-3.66) | <0.001 |
|  | ART*PIH | 1.08 (0.97-1.22) | 0.173 | 1.06 (0.94-1.19) | 0.362 |
| Multipara | ART | 1.06 (0.97-1.15) | 0.204 | 1.20 (1.11-1.31) | <0.001 |
|  | PIH | 3.33 (3.27-3.39) | <0.001 | 3.73 (3.65-3.81) | <0.001 |
|  | ART*PIH | 1.15 (0.98-1.36) | 0.094 | 1.14 (0.96-1.35) | 0.147 |
| **Premature birth** |  |  |  |  |  |
| Total sample | ART | 1.42 (1.37-1.47) | <0.001 | 1.59 (1.53-1.65) | <0.001 |
|  | PIH | 2.63 (2.60-2.66) | <0.001 | 2.61 (2.58-2.65) | <0.001 |
|  | ART*PIH | 0.97 (0.90-1.05) | 0.523 | 0.98 (0.91-1.06) | 0.664 |
| Age<35 years | ART | 1.36 (1.28-1.44) | <0.001 | 1.72 (1.62-1.82) | <0.001 |
|  | PIH | 2.58 (2.55-2.61) | <0.001 | 2.63 (2.59-2.66) | <0.001 |
|  | ART*PIH | 0.97 (0.86-1.11) | 0.692 | 0.98 (0.86-1.12) | 0.783 |
| Age≥35 years | ART | 1.30 (1.24-1.36) | <0.001 | 1.51 (1.44-1.58) | <0.001 |
|  | PIH | 2.81 (2.75-2.88) | <0.001 | 2.58 (2.52-2.65) | <0.001 |
|  | ART*PIH | 0.92 (0.83-1.02) | 0.121 | 0.98 (0.89-1.09) | 0.745 |
| Primipara | ART | 1.45 (1.38-1.53) | <0.001 | 1.53 (1.45-1.61) | <0.001 |
|  | PIH | 2.63 (2.58-2.67) | <0.001 | 2.65 (2.60-2.70) | <0.001 |
|  | ART*PIH | 0.97 (0.88-1.08) | 0.602 | 0.97 (0.88-1.08) | 0.578 |
| Multipara | ART | 1.47 (1.39-1.55) | <0.001 | 1.66 (1.57-1.75) | <0.001 |
|  | PIH | 2.73 (2.69-2.77) | <0.001 | 2.58 (2.53-2.62) | <0.001 |
|  | ART*PIH | 0.99 (0.87-1.12) | 0.870 | 1.01 (0.89-1.16) | 0.844 |
| **NICU admission** |  |  |  |  |  |
| Total sample | ART | 1.57 (1.51-1.64) | <0.001 | 1.40 (1.35-1.46) | <0.001 |
|  | PIH | 2.74 (2.71-2.77) | <0.001 | 2.34 (2.31-2.37) | <0.001 |
|  | ART*PIH | 0.86 (0.79-0.94) | <0.001 | 0.92 (0.85-1.01) | 0.069 |
| Age<35 years | ART | 1.59 (1.49-1.68) | <0.001 | 1.52 (1.43-1.61) | <0.001 |
|  | PIH | 2.70 (2.67-2.74) | <0.001 | 2.35 (2.32-2.38) | <0.001 |
|  | ART*PIH | 0.83 (0.72-0.95) | 0.006 | 0.89 (0.78-1.02) | 0.102 |
| Age≥35 years | ART | 1.42 (1.35-1.49) | <0.001 | 1.40 (1.33-1.47) | <0.001 |
|  | PIH | 2.85 (2.78-2.92) | <0.001 | 2.36 (2.29-2.42) | <0.001 |
|  | ART*PIH | 0.85 (0.77-0.95) | 0.004 | 0.93 (0.83-1.04) | 0.215 |
| Primipara | ART | 1.50 (1.43-1.58) | <0.001 | 1.38 (1.31-1.45) | <0.001 |
|  | PIH | 2.40 (2.36-2.44) | <0.001 | 2.22 (2.18-2.26) | <0.001 |
|  | ART*PIH | 0.94 (0.85-1.04) | 0.219 | 0.97 (0.87-1.07) | 0.533 |
| Multipara | ART | 1.48 (1.39-1.57) | <0.001 | 1.39 (1.31-1.49) | <0.001 |
|  | PIH | 2.96 (2.91-3.01) | <0.001 | 2.48 (2.43-2.52) | <0.001 |
|  | ART*PIH | 0.81 (0.70-0.94) | 0.006 | 0.87 (0.74-1.01) | 0.072 |

Note: ART*PIH, interaction between ART and PIH; OR, odds ratio; 95%CI, 95% confidence interval; crude model, univariate logistic regression model; adjusted model, multivariate logistic regression model adjusted for maternal age at delivery (not in age subgroups), race of mother, educational level of mother, marital status, age of father, race of father, educational level of father, smoking before pregnancy, smoking during pregnancy, total number of prenatal care visits for this pregnancy, pre-pregnancy BMI, pre-pregnancy chronic diabetes, gestational diabetes, clinical chorioamnionitis or maternal fever during labor, previous preterm births, previous cesarean section, parity (not in parity subgroups), and gestational weight gain.
